# Do surgeries have a number needed to treat of 1.0?

**DOI:** 10.1590/1806-9282.20250347

**Published:** 2025-06-16

**Authors:** José Nunes de Alencar, Ana Camila de Castro Gandolfi

**Affiliations:** 1 Instituto Dante Pazzanese de Cardiologia, Division of Research – São Paulo (SP), Brazil.; 2 Universidade Federal de São Paulo, Escola Paulista de Medicina, Department of Neurology and Neurosurgery, Discipline of Neurosurgery – São Paulo (SP), Brazil.

The integration of cognitive biases into clinical reasoning has long been a subtle yet influential factor shaping outcomes in healthcare. A cognitive bias describes subconscious cognitive "shortcuts" employed to navigate complexity and uncertainty in decision-making. While these shortcuts can improve efficiency, they often introduce errors. In surgery, cognitive biases have been linked to delayed operative interventions, unnecessary procedures, intraoperative complications, and delayed recognition of postoperative problems^1^.

Overconfidence bias represents a well-documented cognitive distortion in which a clinician believes their performance or outcomes are consistently better than average. In surgical practice, this bias can manifest in many subtle ways. Consider surgeons who believe their anastomotic leak rates are better than those of their peers. This perception may prompt them to forgo protective measures, such as diverting loops, under the assumption that their technique alone ensures superior outcomes^2^. Although confidence is a fundamental trait for a successful surgeon, overconfidence strays into error when it disregards uncertainty, variance in patient anatomy, or the unpredictable nature of human physiology. Overconfidence can produce a sense of invulnerability and a flawed conviction that each intervention is unequivocally beneficial^3^. Yet overconfidence does not only exist in terms of technical prowess; it can also manifest in distorted interpretations of outcome measures. In this discussion, it takes the form of what might be considered a hidden assumption: that certain surgical interventions have a "number needed to treat" (NNT) of 1.0.

Consider a clinical scenario that illustrates another, more insidious form of overconfidence bias. In an acute emergency setting, a patient arrives with a surgical condition that, from the surgeon's perspective, clearly demands immediate intervention—perhaps an acute abdomen. Without urgent surgery, the patient faces near-certain death^4^. The decision to operate is swift and appears unequivocal. However, this scenario lays the groundwork for cognitive distortion. If the patient dies on the operating table, the outcome is seen as inevitability fulfilled: the patient's underlying condition would have led to death anyway, so the death is attributed to the natural course of the disease. If an intraoperative error clearly contributed, the surgeon may attribute the failure to a technical slip, an isolated event not reflective of the decision to operate itself. On the other hand, if the procedure is technically flawless yet the patient still expires, the surgeon often views this as an unavoidable fate rather than a misinterpretation of risks or an overestimation of benefit.

By contrast, if the patient survives the surgery and leaves the operating room alive, the intervention is considered an undeniable success. The reasoning here is straightforward: without the intervention, death was certain; with the intervention, the patient now has a renewed chance at life. From the surgeon's viewpoint, each successful surgical rescue reinforces the idea that their intervention was singularly lifesaving, justifying the initial decision-making process. Over time, this creates a situation where the surgeon implicitly believes that for conditions perceived as universally lethal without surgery, the NNT is 1.0—one treatment needed to achieve one life saved every time.

Another dimension of this issue arises in the postoperative period, which can fade from the operating surgeon's mental frame. The patient's complex postoperative course—admission to the intensive care unit (ICU), development of sepsis, multiorgan dysfunction, or other delayed complications—may be overlooked in the surgeon's heuristic. The operation's immediate impact overshadows the larger clinical trajectory. Should the patient die hours or days after surgery in the ICU, such a death is often divorced from the initial decision to operate and attributed to complex postoperative variables. Furthermore, it is not uncommon for surgeons to unconsciously make illusory correlations with external events or circumstances to justify a bad result. This fragmentation of the patient's journey can further entrench the overconfidence bias, as the surgeon's memory anchors to the moment of surgical decision-making and immediate operative outcome rather than integrating the entire treatment course into the assessment of success.

This pattern can lead to several distortions. First, surgeons may neglect the possibility that not all presentations without immediate surgical intervention are guaranteed to end in death. Consider acute appendicitis. While prompt surgical intervention (appendectomy) is often warranted, not every patient who presents with right lower quadrant pain and a mild elevation in inflammatory markers faces certain mortality. Conservative management with antibiotics and careful observation has been increasingly supported by research^5^. In many cases, surgery is highly beneficial, but it is not always the only life-saving option—and the risk of surgical complications is not irrelevant. By disregarding this nuance, surgeons may commit more patients to the operating room under the erroneous assumption that it is always absolutely necessary. This reveals an availability bias, in which surgeons offer what is available in their treatment arsenal. Moreover, even for surgeries that can be interpreted by a parachute analogy, the individual risk of complications and death is always present.

Second, ignoring deaths that occur on the operating table as inevitable outcomes of disease—rather than counting them as failings of the intervention—reinforces a biased belief system. If the surgery is considered "no-fault" whenever it fails, then it is never counted as an unsuccessful case. The irony is that this leads to a perception that the NNT is 1.0 because failure modes are systematically dismissed.

Third, excluding postoperative mortality from the mental calculus skews the perceived benefit of the intervention. Postoperative deaths can be complex events involving multiple factors, but they still represent an outcome that stems, at least in part, from the treatment strategy. When postoperative mortalities are filtered out of the decision-making narrative, the surgeon is again left with a distorted sense of efficacy. Surgery is often seen as a magical solution, not only by surgeons but also by other doctors and patients. For elective surgeries, surgery is usually seen as the fastest solution, ignoring the fact that postoperative care and rehabilitation are decisive to the success of the procedure.

**Figure 1:**
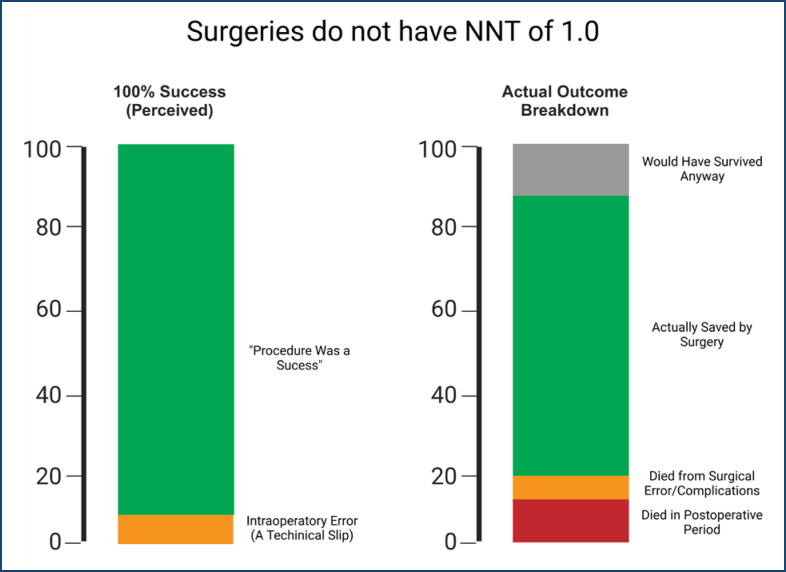
The left bar represents the overconfident perception of a 100% success rate. In contrast, the right bar illustrates a more realistic outcome distribution for a highly effective procedure: 65 of every 100 patients are genuinely saved by surgery (green), leading to an NNT of approximately 1.53 (calculated as 100 ÷ 65). Some individuals would have survived without surgery (gray), some die from surgical errors or complications (orange), and others succumb in the postoperative period (red). Recognizing that the NNT is not 1.0 highlights opportunities to enhance postoperative care and minimize preventable failures.

In truth, no medical intervention—not even emergency surgery—has a true NNT of 1.0. Complex human biology, variability in clinical presentations, patient comorbidities, and the potential for serious complications all mean that the net benefit of any procedure or treatment is never absolute (Figure 1). Overconfidence bias obscures these nuances, potentially driving unnecessary operative interventions and heroic attempts in acute settings without due consideration of alternative strategies or the full continuum of care^6^. Recognition of this bias is essential. By engaging in metacognition, surgeons can critically appraise their implicit assumptions^7^. Approaches could include prospectively tracking all outcomes—including intraoperative and postoperative mortalities—and systematically reviewing both success and failure cases^8^. Morbidity and Mortality Conferences (M & M) are the right place for this type of discussion, where surgeons can review mistakes and figure out what to do differently next time. However, M & M Conferences require an environment in which surgeons feel safe to admit and discuss their own mistakes^2^. Since surgeons often make surgical decisions based on their personal clinical experience^9^, discussions with other surgeons can expand their horizons of possibilities. Emphasizing evidence-based guidelines, solid knowledge of statistics, considering watchful waiting strategies where appropriate, and maintaining open, multidisciplinary discussions about treatment options further help dilute the effect of overconfidence.
